# Supplementary material for: Conditional chemoconnectomics (cCCTomics) as a strategy for efficient and conditional targeting of chemical transmission
Source: eLife. 2024 Apr 30;12:RP91927. doi: 10.7554/eLife.91927 (PMC11060718; doi:10.7554/eLife.91927)
Supplement: Figure 3—source data 3. [file elife-91927-fig3-data3.zip › Figure 3 source data3 part 1.zip!/Figure 3-Source data 3-part1/1175-9-CRISPResso_Report_rtnd62ko.zip!/CRISPResso_on_1175_9/2b.Nucleotide_percentage_quilt_around_sgRNA_gtaggccacgcagtagacca.pdf]

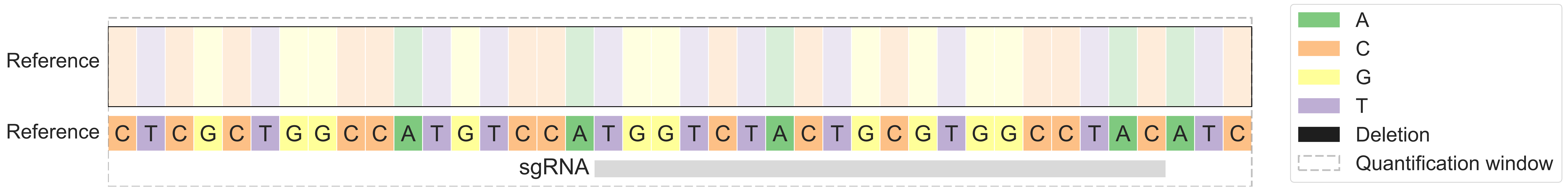

Reference
Reference
C T C G C T G G C C A T G T C C A T G G T C T A C T G C G T G G C C T A C A T C
sgRNA
A
C
G
T
Deletion
Quantification window
